# Diagnosis of COVID-19. What have we learned after two years of the pandemic?

**DOI:** 10.1515/almed-2022-0041

**Published:** 2022-06-15

**Authors:** Melania Iñigo, Gabriel Reina, José Luís Del Pozo

**Affiliations:** Department of Microbiology, Clínica Universidad de Navarra, Madrid, Spain; Department of Microbiology, Clínica Universidad de Navarra, Pamplona, Spain; Institute of Tropical Health Universidad de Navarra (ISTUN), Pamplona, Spain; Navarra Institute for Health Research (IdiSNA), Pamplona, Spain; Infectious Diseases Division, Department of Microbiology, Clínica Universidad de Navarra, Pamplona, Spain

A public health crisis has been threatening the world since the new 2019 coronavirus (2019-nCoV) or severe acute respiratory syndrome coronavirus 2 (SARS-CoV-2) emerged and began to spread in late 2019. As of April 10, 2022, more than 496 million confirmed cases and more than 6 million deaths had been reported in the six WHO regions. However, the total impact of the pandemic has been far greater than reported deaths indicate. Although reported deaths from COVID-19 between January 1, 2020, and December 31, 2021, were 5.94 million worldwide, a recent work estimates that 18.2 million people actually died worldwide due to the COVID-19 pandemic (measured by excess mortality during that period) [1].

Vaccines have significantly reduced COVID-19-related deaths, with more than 10 billion doses having been administered worldwide. However, the distribution of vaccines remains highly unequal. Most of the world’s wealthier countries have vaccinated and even administered booster doses to most of their populations, yet the poorest countries have extremely low vaccination coverage rates. In recent months, we have witnessed a turning point in the pandemic due to the high contagiousness of the new variants, their lower aggressiveness and the fact that a large part of the population is immunized through vaccines or through natural infection. Due to these facts, many local governments have relaxed public health restriction measures. Nevertheless, experience and data suggest that new variants will emerge that will generate new outbreaks and have unpredictable implications for prevention and treatment strategies.

The rapid and accurate diagnosis of SARS-CoV-2 infection has become a priority issue since first cases were reported. Different methods have been developed, with several strengths and limitations depending on the target population, the epidemiological scenario, and the turn-around time. Real-time polymerase chain reaction (RT-PCR) identifies the presence of SARS-CoV-2 RNA in biological samples. It is considered the gold standard diagnostic test due to their high specificity and sensitivity. RT-PCR is based on the specific detection of different regions of the virus genome, mainly structural protein genes, such as spike, envelope, membrane, and nucleocapsid, preferably with simultaneously detection of several viral targets to avoid false-negative results. Although RT-PCR is the gold standard, the need for trained personnel and specific equipment, the cost of technique and the long turn-around time has led to the development of other techniques available to meet the high demand for diagnosis necessary in a pandemic status [2].

Antigen tests (AgT) are immunoassays that detect the presence of a specific viral antigen. These tests can be performed quickly and at the same point of care, with turn-around times less than 30 min and low costs associated. AgT are in general less sensitive than molecular techniques, so these tests are recommended during the first days of symptoms (when viral load is higher), to detect individuals with greater viral load (greater risk of contagion) and in the study of outbreaks in closed environments. While molecular techniques can be excessively sensitive and remain positive for a long-time detecting individual who are no longer contagious, AgT more accurately reflect the patient’s infective capacity so they are widely recommended in the last stage of the infection to end the quarantine of positive cases detected. Furthermore, these tests can only be performed on nasopharyngeal swabs and, as they are qualitative, cannot quantify the amount of antigen present [3, 4]. Unlike AgT, molecular techniques allow semi-quantification of the viral load present in the sample using the specific threshold for Ct value. Higher Ct values represent a low viral RNA load and a lower risk of infection transmission (Ct>33–34 seem to be no longer infectious); this may correspond to an incubation period or convalescent stage, or to primary replication of the virus at other sites in the body (lower respiratory tract). In contrast, low Ct values correspond to a high viral load, correlated with high infection risk. Although limited data can establish the relation between the patient’s viral loads and prognoses, such as disease progression or death rate, several authors reported an inverse correlation between SARS-CoV-2 Ct value and mortality. It is important to note that Ct values are commonly affected by pre-analytic (collection technique, transport and storage conditions before the testing…), analytic (viral RNA load in the collected samples, primer design, reagents used…), and post-analytical (mainly results interpretation) variables. Therefore, the standardization of all these variables is essential for an appropriate interpretation of Ct values [5].

Finally, it is described the continuous appearance of new viral variants because of numerous gene mutations from the original Wuhan-strain. The CDC and the WHO have classified some of these new strains as variants of concern (VOC). These mutations may affect important viral functional characteristics, including infectivity and an enhanced ability to evade natural infection and/or vaccine-induced neutralizing antibody responses. Therefore, it is a matter of concern since these new variants could evade immune responses from previous vaccination and or infection, or even select drug-resistant mutants. Although next-generation sequencing (NGS) is considered the gold standard for SARS-CoV-2 variant identification and characterization of mutations in the viral genome, it is a complex technique, only available to the most specialized laboratories. Multiple commercial easy RT-PCR kits have been developed for the study of these new variants, allowing faster and more representative typing of circulating strains. NGS allows confirm RT-PCR results and monitor the emergence and dynamics of novel SARS-CoV-2 variants. Considering the dynamics of viral mutation and the speed with which new VOC emerge, continuous surveillance of variants is mandatory [6].

On the other hand, the detection of antibodies against SARS-CoV-2 allows the identification of specific markers associated with vaccine response (anti-spike antibodies), since most vaccines are based on the Spike protein. To differentiate the population that has undergone a natural infection, anti-nucleoprotein antibodies (anti-N) can also be detected in serum. The quantification of anti-spike allows assessing the degree of response to vaccination and can confirm an infection; if an increase is detected without vaccine boosting and/or anti-N seroconversion is observed. Similarly, reinfection can be confirmed by evaluating increases in antibody levels against these two targets. A WHO international standard (NIBSC code 20/136) has been developed whose values are expressed as binding antibody units (BAU) per mL. The level of 143 BAU/mL of antibodies has been proposed as an adequate level of protection against infection [7], but this can be highly variable depending on the circulating VOC and, therefore, it should not be the exclusive marker used to assess the need for vaccine boosting.

The presence of neutralizing antibodies is related to protection against SARS-CoV-2 virus infection. However, the circulation of VOCs such as Omicron BA.1 or BA.2, with a high rate of mutations in the Spike protein, reduces the protective capacity of these antibodies generated by the vaccine or by previous infections [8]. In addition to neutralization, the importance of Fc-mediated action of non-neutralizing antibodies has been recently highlighted to protect from severe progression by induction of macrophages and NK cells. Moreover, this protective effect among vaccinated individuals or after natural infection would not be so dependent on the circulating VOC [9].

Both vaccines and natural infection by SARS-CoV-2 or similar coronaviruses, induce the generation of specific T cells with effector capacity and immunological memory. The action of T cells is essential to reduce the severity of the infection and facilitate the recovery of the infected individual. In addition, its protective effect can last for years and does not depend on the infecting VOC, as there is a cross-protective effect induced by different coronaviruses [10].

The evaluation of specific T cells is more expensive, labor intensive and time consuming than serology, since it requires the stimulation of viable cells with specific SARS-CoV-2 antigens, and the subsequent measurement of cytokines produced (IFN gamma, IL-2). The reference method requires the separation of PBMC cells by Ficoll gradient and study by flow cytometry. However, other tests have been developed using whole blood, suitable for their use in the clinical lab and giving valid results to evaluate the specific cellular response [11]. These methods, called interferon gamma release tests (IGRA test), have been marketed by different companies and allow a rapid determination of whether or not there is an effector cellular immune response after vaccination or infection. To do this, the individual’s blood is stimulated with antigens of the spike protein or the complete genome of the virus and subsequently the production of interferon gamma is quantified.

The different methods available allow the detection of specific T cells, which eventually reduce the chance of serious illness. In addition, T-cell activity is preserved longer than antibodies after an infection or vaccination. Even, the Omicron can be target of T cells generated by different vaccines or unvaccinated convalescent COVID-19 patients. It has been recently reported that they maintained 70–80% of the CD4+ and CD8+ response to Omicron spike and the magnitude of cross-reactive T cells was similar for Omicron, Beta, and Delta variants as T cells can recognize more sites along the spike protein than can antibodies [12].

To summarize, unless vaccines are reformulated to improve the production of specific antibodies against new variants, those individuals with specific T cells following vaccination or natural infection will get little benefit from booster shots. Therefore, the assessment of T-cell activity may be a suitable tool to personalize the need for further boosting, particularly among vulnerable populations who may be at increased risk of severe disease.
